# Predicting high-cost healthcare users: development and external validation of multivariable models using the HUNT and Tromsø studies linked to Norwegian health registries

**DOI:** 10.1186/s12913-026-14295-7

**Published:** 2026-03-06

**Authors:** Bjørnar Berg, Steven Hicks, Vajira Thambawita, Tarjei Rysstad, Qiuzhe Chen, Margreth Grotle

**Affiliations:** 1 Centre for Intelligent Musculoskeletal Health, Faculty of Health Sciences, Oslo Metropolitan University, Pilestredet 50, Oslo, 0167 Norway; 2 Department of Holistic Systems SimulaMet, Oslo, Norway; 3 Sydney Musculoskeletal Health, Faculty of Medicine and Health, The University of Sydney, Camperdown, New South Wales Australia; 4 Department of Research and Innovation, Division of Clinical Neuroscience, Oslo University Hospital, Oslo, Norway

**Keywords:** Prediction, Healthcare utilisation, Health expenditures, High-cost users

## Abstract

**Background:**

Rising healthcare costs and demand call for better identification of individuals at risk of high-cost healthcare use. Few prediction models use detailed survey data or address persistent high-cost use in the general population. This study aimed to develop and externally validate prediction models for all-cause single-year and persistent high-cost healthcare use, and to assess whether adding survey data to administrative registry data improved performance.

**Methods:**

This was a prognostic study based on two population-based cohorts, the Trøndelag Health Study (HUNT4; model development) and the Tromsø Study (Tromsø7; external validation), linked to prospectively collected health registry data from primary and secondary care. Outcomes were (1) single-year high-cost use, defined as being in the top 25% of total healthcare costs in year one after survey completion, and (2) persistent high-cost use, defined as being in the top 25% in both years one and two. Predictors included self-reported sociodemographic and health-related variables and health registry data (prior-year costs and a morbidity index). Logistic regression models were developed for each outcome and internally validated via five-fold cross-validation. Model performance was assessed through discrimination and calibration. XGBoost models were trained and tested for benchmarking. External validation applied the developed models without refitting. We also developed and validated registry-only and survey-only models to compare performance against the full model.

**Results:**

The development cohort included 42,049 individuals, and the external validation cohort included 20,942. In internal validation, the full logistic regression model achieved C-statistics of 0.79 (95% CI 0.78–0.79) for single-year high-cost use and 0.83 (95% CI 0.83–0.84) for persistent high-cost use. Corresponding C-statistics in external validation were 0.78 (95% CI 0.77–0.78) and 0.82 (95% CI 0.81–0.83). The models appeared well-calibrated on calibration plots. Full models showed significantly higher C-statistics than registry-only models (*p* < 0.001).

**Conclusion:**

Prediction models for identifying all-cause single-year high-cost and persistent high-cost healthcare use in the general adult population were developed and validated, showing good discrimination and calibration. The models can inform targeted preventive strategies and population health management. Incorporating self-reported survey data improved predictive performance, supporting the use of combining data sources for risk stratification.

**Supplementary Information:**

The online version contains supplementary material available at 10.1186/s12913-026-14295-7.

## Background

Rising healthcare costs and increasing demand for services are straining health systems worldwide [1]. A critical challenge lies in the fact that relatively small segments of the population consistently account for a large share of overall healthcare expenditures [2, 3]. Identifying these high-cost healthcare users has become increasingly important as we face ageing populations and growing financial pressure. A sustainable healthcare system depends not only on meeting the needs of current high-cost users but also on identifying and mitigating future high-cost use at the population level [4, 5].

Although many individuals experience increases in healthcare use due to acute illness or injury, a subset persist as high-cost users over time [6]. These persistent high-cost patients often have complex care needs related to multimorbidity, mental health, or social vulnerability [6]. Understanding who is likely to become or remain a high-cost user is essential for designing targeted interventions, improving care coordination, and optimising resource allocation [5, 7]. Different definitions of high healthcare use have been applied in prior research, typically based on relative thresholds ranging from the top 5% to top 25% of annual costs or utilisation distribution [7]. While narrower thresholds tend to be dominated by individuals with acute or severe conditions, broader thresholds capture a more heterogeneous population with sustained high utilisation.

Several models have been developed to predict high-cost healthcare users in the general population using administrative claims data alone [6, 7]. However, few studies have combined detailed self-reported survey data with health registry data in European settings. Models incorporating population-based survey data have been developed in countries such as Canada and Japan [8, 9]. Osawa and colleagues demonstrated that while models using only clinical data from national screening programs in Japan achieved acceptable discriminatory performance, adding clinical variables to prior-year healthcare cost data resulted in only marginal improvements [9]. In contrast, self-reported health data improved the ability to identify individuals at risk of becoming high-cost users beyond models using health registry data alone in studies of US employees [10, 11]. However, the potential added value of incorporating detailed self-reported health, lifestyle, and psychosocial information remains insufficiently explored in the general population in publicly funded healthcare systems with universal access to services. Improved prediction of high-cost users within this setting could help inform the development of targeted care management strategies and support healthcare planning.

Extending this prior work, we aimed to develop and externally validate multivariable prediction models for high-cost healthcare use in the general population using administrative healthcare registry data and self-reported data from two large, population-based health surveys in Norway. Specifically, we aimed to develop and externally validate models for (1) single-year high-cost healthcare use (first year after baseline), and (2) persistent high-cost healthcare use (first and second year after baseline). We also compared the performance of a full model that included both prior-year healthcare cost and self-reported survey data, with a model that used only prior cost data alone.

## Methods

### Study design

Prediction models were developed and externally validated using a prospective cohort design. Data were drawn from the fourth wave of the Trøndelag Health Study (HUNT) [12] for model development, and the seventh survey of the Tromsø Study for external validation, both linked to administrative healthcare registry data. The study is reported in accordance with the Transparent Reporting of a multivariable prediction model for Individual Prognosis or Diagnosis (TRIPOD+AI) guideline [13].

This study is part of the AID-Spine project, which has been approved by the regional ethics committee of the Health Region of South-East Norway (2022/371282) and the Norwegian Social Science Data Service (558480). All survey participants gave written informed consent upon participation.

### Patient population and data sources

The HUNT study is a population-based cohort study of adults aged 20 years and older in Trøndelag County, central Norway [12]. We used data from HUNT4, conducted between 2017 and 2019, including all participants. The Tromsø Study is a multipurpose health survey conducted in the largest municipality in northern Norway [14]. Tromsø7 was conducted in 2015–2016, with all inhabitants aged 40 years and above invited to participate, and we included all participants in the current study. Survey completion date served as the index date for cohort entry and the start of follow-up. The unique identification number allocated to all Norwegian residents allowed linkage to national healthcare registry data.

### Outcomes

Healthcare costs during the 24 months following survey participation were identified in the Norwegian Control and Payment of Health Reimbursements Database (KUHR) for primary care and the Norwegian Patient Registry (NPR) for specialist care [15]. The KUHR holds data on reimbursement claims from general practitioners, physiotherapists, chiropractors, and emergency room clinicians. The NPR covers activity data from all specialist health services, including consultations and admissions in government-owned hospitals and outpatient clinics and private contract specialists eligible for government reimbursement. All-cause healthcare utilisation was identified within the 0 to 24-month follow-up period by calculating the time difference between the date of each healthcare contact and the survey completion date. Costs related to face-to-face consultations and indirect contacts (e.g., telephone conversations, prescriptions, electronic communication) were included as a measure of the total burden on the healthcare system. Annual costs were summarised at the individual level and adjusted to 2022 price levels. Two different outcomes were defined: *single-year high-cost healthcare use*, defined as being in the top 25% of total healthcare costs in year one after survey completion; and *Persistent high-cost healthcare use*, defined as being in the top 25% in both years one and two after survey completion. The top 25% threshold was chosen because it reflects a broader segment of the population with sustained high utilisation, representing groups that may require coordinated care or preventive strategies, while also providing sufficient outcome prevalence for stable model estimates. Sensitivity analyses using the top 10% as the cut-off were conducted to assess the robustness of the main analyses.

### Predictors

We included common predictors (*n* = 29) from HUNT4 and Tromsø7, covering sociodemographic characteristics, health-related behaviours and lifestyle factors, and self-reported health. All predictors and their response categories are detailed in Supplementary Table 1. Two predictors from the administrative health registries were included: the quartile of total healthcare costs during the 12 months preceding survey participation, and a morbidity index based on the International Classification of Primary Care (ICPC-2) codes from primary care [16].

### Sample size

The sample size was restricted to the number of participants in the health surveys. To assess whether the available sample size was sufficient for model development and validation, we conducted a priori sample size calculations [17, 18]. Given an event rate of 25%, an assumed C-statistic of 0.82 [9], and 63 predictor parameters, a minimum sample size of 2065 (517 events) would be required for model development. For model validation, a minimum sample size of 3067 (767 events) would be required when targeting a confidence interval width of 0.2 for the calibration slope and 0.1 for the C-statistic. The calculation was based on an event rate of 25%, a C-statistic of 0.79, and a normally distributed linear predictor with a mean of −1.41 (standard deviation 1.08), all derived from the developed model. Both the development and external validation cohort substantially exceed the minimum required sample size for regression-based prediction models. The pmsampsize and pmvalsampsize packages in Stata were used for the calculations [17, 18].

### Data cleaning

To guarantee uniformity in the evaluation of identical constructs and to synchronise response options, an in-depth analysis was performed to identify equivalent predictors across the two datasets. Minor adjustments were made to some categorical variables as required to ensure uniformity in the number of response categories (Supplementary Table 1). Data quality assessments were also conducted during data cleaning and linkage, including assessment of missing values and cross-referencing participant identifiers across the two health surveys to ensure each participant was unique to a survey. No data were missing for healthcare utilisation, as we used administrative registry data routinely collected during the participants’ interactions with healthcare services [15].

### Statistical analysis

Descriptive data for baseline characteristics were calculated separately for the two cohorts. To illustrate the distribution of healthcare costs, Lorenz curves were constructed for each cohort [19]. Missing data on predictors were assumed to be missing at random and handled using multiple imputation by chained equations, generating 20 imputed datasets [20]. All predictors and outcome data were included in the imputation models. Model performance was assessed within each imputed dataset, and estimates were pooled using Rubin`s rule [21]. Discrimination (C-statistic) and calibration (calibration-in-the-large [CITL] and calibration slope) with 95% confidence intervals (95% CI) were evaluated in the internal validation of the developed models and the external validation.

Separate logistic regression models were developed for the two outcomes (single-year high-cost and persistent high-cost). Multivariable fractional polynomials were applied to account for potential non-linear relationships, and all predictors were forced into the models without automated variable selection. Model performance was internally validated using 5-fold cross-validation and closed-form uniform shrinkage was applied to adjust for potential overfitting. To benchmark performance, we trained an XGBoost model using the same sets of predictors. Hyperparameters were optimised using Bayesian optimisation with five-fold cross-validation to identify the best-performing settings for each model (Supplementary Table 2). External validation was performed by applying the developed models directly to the external validation cohort. All internal parameters were fixed, and the models were applied as-is without re-fitting. In addition to the full models combining health registry and survey data, we developed and validated models based on registry-only and survey-only predictors to compare relative performance. The registry only model included prior healthcare costs, the morbidity index [16], age, and sex. DeLong`s test for correlated C-statistics was used to compare the discriminatory performance of the full and registry-only models [22].

We also report calibration plots comparing observed and predicted probabilities, and risk distribution plots illustrating the distribution of predicted probabilities by outcome category [23]. Decision curve analysis was conducted to evaluate clinical usefulness of the full model, survey-only model, and registry-only model [24].

To explore potential variations in model performance by sex, performance metrics were computed separately for male and female subgroups using individual-level predictions. Sensitivity analyses were also conducted using an alternative outcome definition based on the top 10% of total healthcare costs to assess the robustness of the main analyses.

Data cleaning, multiple imputation, and logistic regression modelling were conducted using Stata version 18.0. The machine learning analyses were performed using Python version 3.8.13. Analysis code is available in repository https://github.com/bjornarb88/AID-Spine-HCU-risk-models.

### Patient involvement

A patient representative who regularly participates in research meetings as part of the AID-Spine project contributed to discussions on the study aims, design, and interpretation of results.

## Results

The development cohort included 42,049 individuals (43% male; mean age 55.8 ± 17.0 years), and the external validation cohort included 20,942 individuals (53% male; mean age 57.3 ± 11.0 years) (Table 1). Participants in the external validation cohort were more likely to hold a university degree and had a higher prevalence of chronic pain in all body regions, despite reporting lower overall pain intensity. A total of 10,513 (25%) and 5236 (25%) individuals were classified as single-year high-cost users in the development and validation cohorts, respectively. The corresponding numbers for persistent high-cost use were 5607 (13.3%) and 2829 (13.5%). Missing data on predictors were minimal in both cohorts (Supplementary Table 3).

**Table 1:** Descriptive characteristics of the development cohort and external validation cohort

	Development cohort (*n* = 42,049)	External validation cohort (*n* = 20,942)
Sex, Male	17,985 (43)	11,014 (53)
Age (years)	55.8 ± 17.0	57.3 ± 11
Marital status		
Unmarried	11,375 (27)	4,977 (24)
Married	23,201 (55)	11,726 (56)
Widow(er)	2,882 (7)	1,041 (5)
Divorced	4,510 (11)	3,198 (15)
Body mass index (kg/m^2)	27.3 ± 4.7	27.3 ± 4.5
Pulse (beats/minute)	70.9 ± 12.9	67.2 ± 11.7
Syst. blood pressure (mmHg)	129.3 ± 18.6	129.6 ± 19.8
Dias. blood pressure (mmHg)	73.5 ± 10.1	75.4 ± 10.1
Waist circumference (cm)	97.2 ± 14.2	95.2 ± 13.0
Hip circumference (cm)	101.1 ± 7.4	104.0 ± 8.6
Education		
Primary school	11,424 (27)	4,753 (23)
Upper secondary school	13,751 (33)	5,722 (28)
University < 4 years	8,620 (21)	3,978 (19)
University 4 years or more	8,031 (19)	6,121 (30)
Current health		
Poor	576 (1)	1,140 (6)
Not so good	9,216 (22)	5,395 (26)
Good	24,683 (60)	11,179 (54)
Very good	6,993 (17)	3,048 (15)
Life satisfaction		
Very satisfied	6,259 (15)	5,178 (26)
Satisfied	16,122 (39)	6,497 (32)
Somewhat satisfied	13,074 (31)	4,773 (24)
Neither satisfied nor dissatisfied	5,060 (12)	2,229 (11)
Somewhat dissatisfied	683 (2)	894 (4)
Dissatisfied or very dissatisfied	401 (1)	748 (4)
Chronic pain elbows/hands	9,049 (22)	7,110 (36)
Chronic pain neck	14,201 (35)	10,155 (51)
Chronic pain upper back	4,823 (12)	4,962 (26)
Chronic pain lumbar back	10,677 (26)	8,163 (42)
Chronic pain hip/leg	14,441 (35)	8,857 (45)
Pain intensity		
No pain	10,532 (26)	6,849 (33)
Mild	14,808 (36)	10,122 (49)
Moderate	11,714 (28)	2,765 (14)
Severe	3,857 (9)	698 (3)
Extreme	473 (1)	72 (0)
Spec. consultation last 12 mo.	5,099 (13)	4,157 (21)
Admitted hospital last 12 mo.	5,396 (13)	2,255 (11)
Alt. med. consultation last 12 mo.	2,007 (5)	1,382 (7)
Smoking status		
Never smoked	18,392 (44)	7,911 (38)
Ex-occasional smoker	3,912 (9)	715 (3)
Current occasional smoker	518 (1)	825 (4)
Ex-daily smoker	15,595 (37)	8,462 (41)
Current daily smoker	3,440 (8)	2,877 (14)
Alcohol frequency		
Never	4,617 (11)	1,675 (8)
Monthly or less	11,090 (27)	5,105 (25)
2–4 times a month	17,301 (42)	7,848 (38)
2–3 times a week	7,164 (17)	4,949 (24)
4 times or more a week	1,321 (3)	1,237 (6)
Headache last 12 mo.	15,315 (38)	6,329 (31)
HUNT act. index (range 0–15)	3.2 ± 2.9	3.4 ± 2.9
Type of work		
Not working	16,570 (40)	6,891 (34)
Mostly sedentary	8,892 (22)	7,829 (38)
Requires a lot of walking	7,683 (19)	3,206 (16)
Requires you to walk and lift	6,808 (16)	2,198 (11)
Heavy manual labour	1,462 (4)	311 (2)
Works shift	5,453 (13)	2,068 (10)
HADS depression (range 0–21)	3.3 ± 2.9	2.8 ± 2.7
HADS anxiety (range 0–21)	4.4 ± 3.5	3.3 ± 2.9
ICPC morbidity index	0.14 ± 0.39	0.11 ± 0.34
Costs preceding 12 mo.		
Quartile 1	588 ± 572	662 ± 396
Quartile 2	3675 ± 1223	3014 ± 1033
Quartile 3	10801 ± 3447	8764 ± 2595
Quartile 4	72142 ± 70012	61191 ± 65970

Values are frequencies (%) or mean ± standard deviation

Syst = systolic; Dias = diastolic; Spec = specialist; Alt. med.=Alternative medicine; HADS = Hospital Anxiety and Depression Scale

**Fig. 1:**
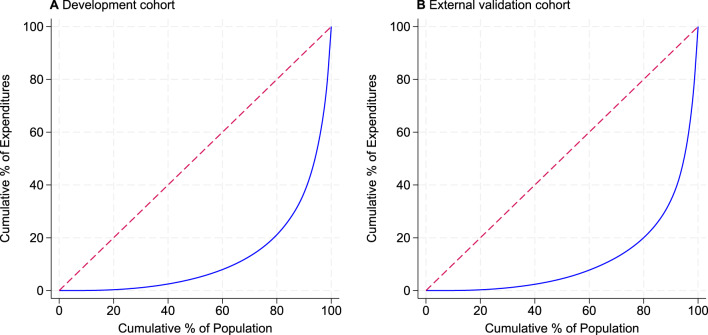
Lorenz curve of healthcare expenditures in the (**A**) development cohort and (**B**) external validation cohort. The blue curve shows the cumulative percentage of healthcare expenditures accounted for by the cumulative percentage of individuals. The dashed red line represents perfect equality

In the first year of follow-up, the median annual healthcare cost was 6438 NOK (671 USD; exchange rate: 9.6 NOK per US dollar, 2022) in the development cohort and 5820 NOK (606 USD) in the external validation cohort. Healthcare costs were highly concentrated, with the top 25% of individuals accounting for 83.5% of total costs in the development cohort and 84.2% in the validation cohort (Fig. 1).

### Single-year high-cost prediction

**Fig. 2:**
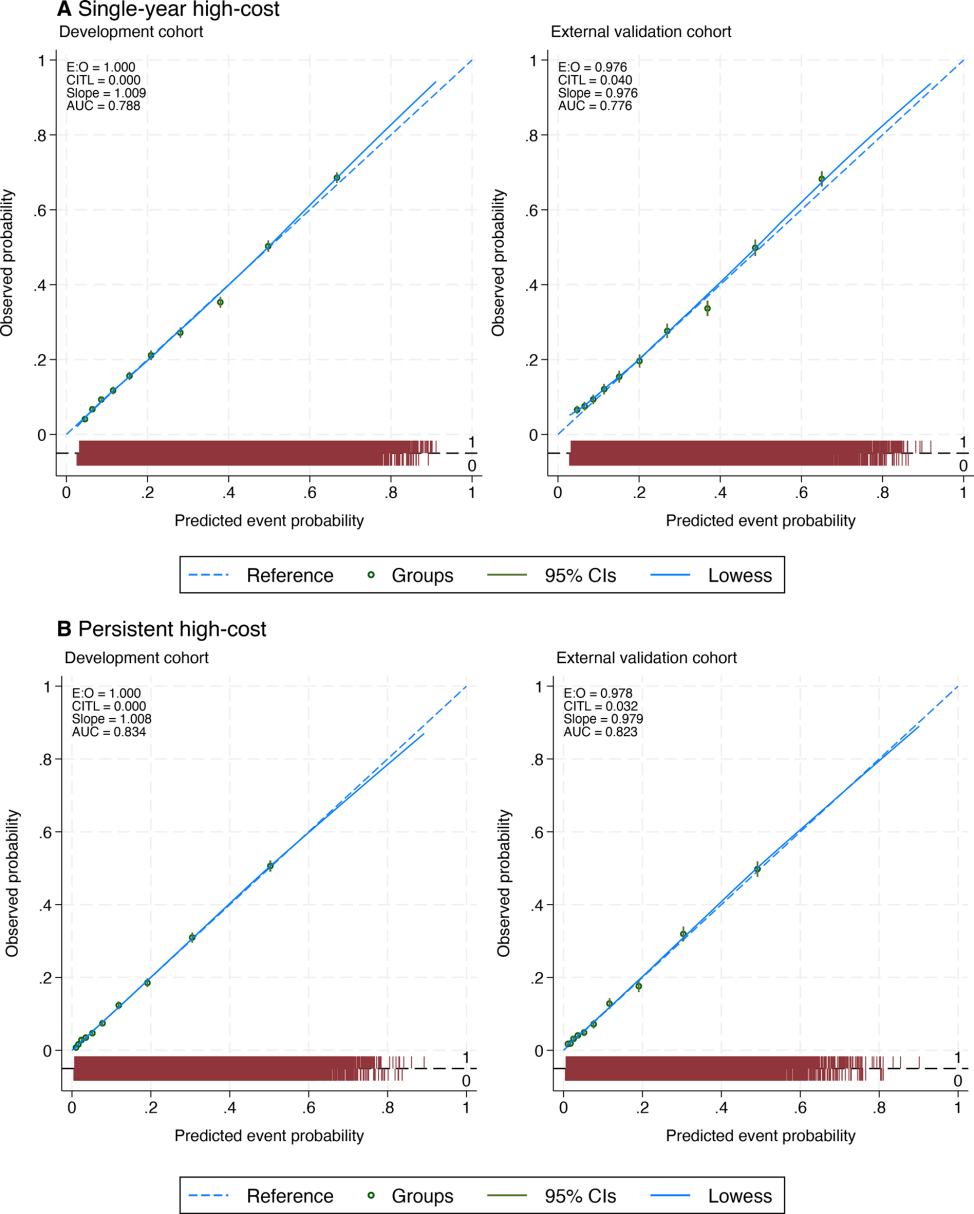
Calibration plots for the development and external validation cohorts for (**A**) single-year high-cost healthcare use and (**B**) persistent high-cost healthcare use

**Fig. 3:**
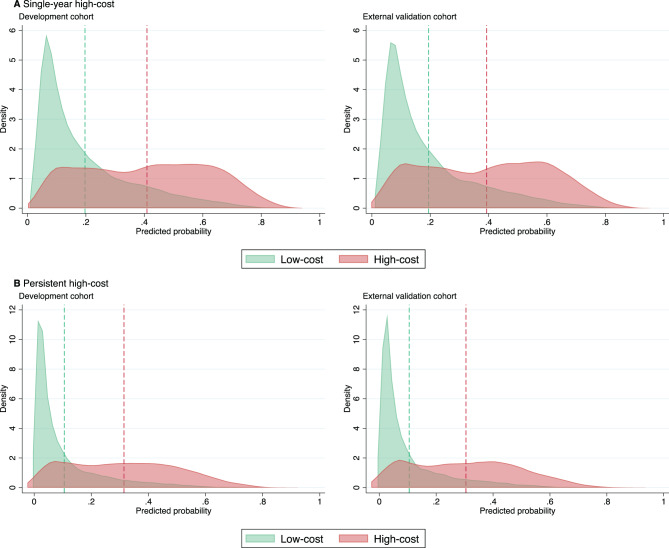
Distribution of predicted probabilities in the development and external validation cohorts for (**A**) single-year high-cost healthcare use and (**B**) persistent high-cost healthcare use. Vertical dashed lines indicate the mean predicted probabilities for each outcome group

Models predicting single-year high-cost use showed good discrimination and calibration, with consistent performance across development and external validation. In the development cohort, the internally validated logistic regression model predicting single-year high-cost use achieved a C-statistic of 0.79 (95% CI: 0.78 to 0.79) and excellent calibration (Table 2). Model performance remained consistent in the external validation cohort, with a C-statistic of 0.78 (95% CI: 0.77 to 0.78), CITL of 0.03 (95% CI: −0.02 to 0.08), and calibration slope of 0.98 (95% CI: 0.94, 1.01). Calibration plots are presented in Fig. 2A. Risk distribution plots showed a marked separation of predicted probabilities between outcome groups (Fig. 3A). Important predictors of single-year high-cost use were prior-year healthcare costs, greater morbidity burden, higher pain intensity, and poor self-rated current health. Model coefficients and odds ratios are presented in Supplementary Table 4. The XGBoost models did not improve discriminatory performance (Table 2).

**Table 2:** Model performance for predicting single-year high-cost and persistent high-cost patients using survey and registry data

	Development cohort (n = 42,049)			External validation cohort (n = 20,942)		
	C-statistic	CITL	C-slope	C-statistic	CITL	C-slope
**Single-year high-cost**						
Logistic regression	0.79 (0.78, 0.79)	0.00 (−0.03, 0.03)	1.01 (0.99, 1.03)	0.78 (0.77, 0.78)	0.03 (−0.02, 0.08)	0.98 (0.94, 1.01)
XGBoost	0.78 (0.78, 0.79)	0.01 (−0.02, 0.03)	1.15 (1.13, 1.18)	0.77 (0.76, 0.78)	0.02 (−0.01, 0.06)	1.16 (1.12, 1.20)
**Persistent high-cost**						
Logistic regression	0.83 (0.83, 0.84)	0.00 (−0.03, 0.03)	1.01 (0.99, 1.03)	0.82 (0.81, 0.83)	0.03 (−0.02, 0.07)	0.97 (0.93, 1.01)
XGBoost	0.83 (0.82, 0.83)	−0.07 (−0.10, −0.04)	1.56 (1.52, 1.60)	0.82 (0.81, 0.83)	−0.05 (−0.09, −0.01)	1.54 (1.49, 1.60)

CITL = Calibration-in-the-large; C-slope = Calibration slope

The discriminatory performance of the full model (using survey and registry data) was significantly higher than the reduced model using registry data alone, at both internal and external validation (Table 3). At internal validation, the registry-only model achieved a C-statistic of 0.770 (95% CI: 0.765 to 0.776) compared to 0.788 (95% CI: 0.783 to 0.793) for the full model (*p* < 0.001). Discrimination was also acceptable for models using only survey data, with C-statistics of 0.736 (95% CI: 0.730 to 0.741) at internal validation and 0.716 (95% CI: 0.708 to 0.724) at external validation. Model coefficients and odds ratios for the survey-only and registry-only models are presented in Supplementary Tables 5 and 6, respectively.

**Table 3:** Discriminatory performance in the development and external validation cohorts for predicting single-year and persistent high-cost healthcare use with the full model and reduced models based on survey data or registry data alone

	Development cohort (n = 42,049)				External validation cohort (n = 20,942)			
	Using survey and registry data	Using only survey data	Using age, gender, and registry data	*p*-value*	Using survey and registry data	Using only survey data	Using age, gender, and registry data	*p*-value*
**Single-year high-cost**								
Logistic regression	0.788 (0.783, 0.793)	0.736 (0.730, 0.741)	0.770 (0.765, 0.776)	<0.001	0.776 (0.768, 0.783)	0.716 (0.708, 0.724)	0.765 (0.757, 0.777)	<0.001
XGBoost	0.781 (0.775, 0.786)	0.730 (0.725, 0.736)	0.758 (0.753, 0.764)	<0.001	0.770 (0.763, 0.778)	0.705 (0.697, 0.713)	0.751 (0.744, 0.760)	<0.001
**Persistent high-cost**								
Logistic regression	0.834 (0.829, 0.840)	0.776 (0.770, 0.783)	0.815 (0.809, 0.820)	<0.001	0.823 (0.814, 0.831)	0.760 (0.750, 0.769)	0.809 (0.801, 0.817)	<0.001
XGBoost	0.828 (0.822, 0.834)	0.774 (0.768, 0.781)	0.807 (0.801, 0.813)	<0.001	0.816 (0.808, 0.825)	0.756 (0.747, 0.766)	0.802 (0.793, 0.811)	<0.001

Values are C-statistics with 95% confidence intervals

DeLong`s test comparing the C-statistic of the full model (survey and registry data) with the reduced model (age, gender, and registry data)

**Fig. 4:**
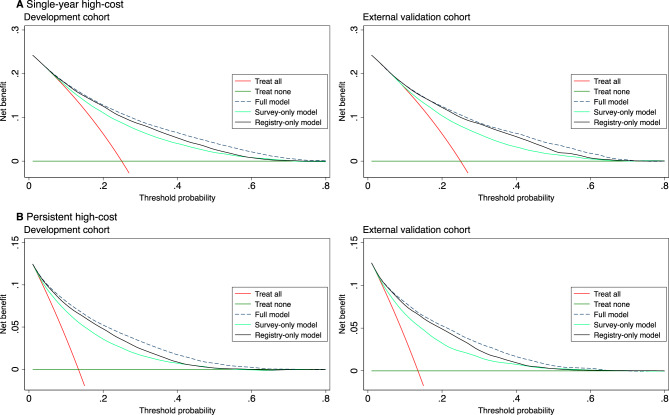
Decision curve analysis in the development and external validation cohorts for (**A**) single-year high-cost healthcare use and (**B**) persistent high-cost healthcare use. “Treat all” represents offering targeted intervention to all individuals, while “treat none” represents offering it to none

Decision curve analysis indicated differences in clinical usefulness between the models, with the full model showing the highest net benefit across a wide range of threshold probabilities (Fig. 4A). Compared to the “treat all” alternative, which represents offering targeted intervention to every individual regardless of risk, the full model showed higher benefit from threshold probabilities of approximately 0.10 onwards. The higher benefit of the full model compared to the registry-only model further supports the added clinical value of incorporating survey data for risk stratification. While the survey-only model yielded lower net benefit, it still outperformed the default strategies of treating all or treating none across multiple thresholds.

### Persistent high-cost prediction

Prediction of persistent high-cost use demonstrated robust performance, with logistic regression models maintaining high discrimination and good calibration in both cohorts. The internally validated C-statistic was 0.83 (95% CI 0.83 to 0.84). External validation showed comparable results, with a C-statistic of 0.82 (95% CI: 0.81 to 0.83), a CITL of 0.03 (95% CI: −0.02 to 0.07), and a calibration slope of 0.97 (95% CI: 0.93 to 1.01). Calibration plots are shown in Fig. 2B. Predicted risk distributions showed a clear separation between outcome groups (Fig. 3B). In addition to high healthcare costs in the previous year, important predictors of persistent high-cost use included greater morbidity burden, higher pain intensity, poor self-reported health, and lower life satisfaction (Supplementary Table 4). XGBoost models offered no meaningful improvement in discriminatory performance and showed signs of miscalibration (Table 2).

The full model (including survey and registry data) outperformed the reduced model with registry data only across both internal and external validation (*p* < 0.001). Models based solely on survey data also demonstrated good discrimination, with C-statistics of 0.776 (95% CI: 0.770 to 0.783) and 0.760 (95% CI: 0.750 to 0.769) in the development and validation cohorts, respectively. The clinical usefulness of the models was generally consistent with the single-year high-cost models, demonstrating similar patterns of net benefit across models in decision curve analysis (Fig. 4B).

### Additional analyses

Sex-stratified analyses showed comparable discrimination and calibration across males and females (Supplementary Table 6). Sensitivity analyses using an alternative 10% high-cost threshold yielded results that were broadly similar to the main analyses (Supplementary Table 7).

## Discussion

We developed and externally validated multivariable models to predict single-year high-cost and persistent high-cost healthcare use in the general population, using self-reported survey data linked to healthcare registry data from primary and secondary care. The models demonstrated strong performance in internal validation, with C-statistics ranging from 0.79 to 0.83 and good calibration across outcomes. Model performance remained stable when applied to an independent external cohort, supporting the generalizability of our findings. Prior-year healthcare costs, morbidity burden, pain intensity, and self-reported health were key predictors across both single-year and persistent high-cost use models. Adding self-reported survey data to registry-based models improved discriminatory performance. However, no meaningful improvement in predictive performance was found with XGBoost compared to logistic regression.

Our models were developed and validated in the general adult population, enhancing their relevance for broader population health management compared to previous prediction models that have focused on specific subpopulations [25–27]. Compared to earlier models predicting high-cost healthcare use in the general population, our models demonstrated similar or stronger discriminatory performance [8, 9, 28–31]. In contrast to these studies, which defined high-cost users using narrower thresholds such as the top 5% or top 1% of spenders, we applied a broader definition based on the top 25% of healthcare spenders. While this approach may yield lower specificity than narrow cut-offs, it allows for the identification of individuals who may be on a trajectory toward very high healthcare costs and may provide a more actionable target group for early and coordinated care interventions. Notably, interventions aimed at the most complex and costly patients have shown limited effectiveness in reducing utilisation and costs, likely due to the multifactorial nature of their health needs [32]. We complemented this approach by developing models to identify persistent high-cost users over two consecutive years. This represents a relatively underexplored yet important area for long-term care planning and management [7].

The prediction models could support healthcare systems by enabling earlier identification of individuals who may benefit from coordinated care, targeted follow-up, or preventive interventions. At the population level, the models could assist healthcare planners in forecasting care needs, prioritising resources, and designing risk-stratification approaches to managing high-cost patients. Assuming the availability of effective follow-up interventions in the future, our decision curve analysis indicated that using the models for risk stratification would yield greater benefit than the alternative strategies of intervening on none or all [33]. Specifically, the full model showed net benefit at threshold probabilities from approximately 0.10. Although the appropriate threshold for targeted follow-up must be determined by decision-makers based on resource constraints and the trade-off between missed cases and unnecessary interventions, thresholds around 0.20 may be pragmatically relevant for healthcare planners.

While most prediction models for high-cost healthcare use rely solely on administrative registry data, there is growing recognition of the potential value of incorporating self-reported and clinical survey data. We found that adding self-reported survey data to registry-only models improved predictive performance across both single-year and persistent high-cost outcomes. While a study from Japan reported limited added value of adding clinical variables to prior-year healthcare costs [9], modest improvements in discrimination have been shown by supplementing administrative health registry data with self-reported information. Boscardin et al. [10] developed a prediction model for identifying high-cost users (top 10%) and reported an increase in the C-statistic from 0.63 to 0.70 when self-reported health, activity limitations, and prior-year utilisation were included alongside administrative health data. Although the discrimination was only acceptable, it highlights the potential for self-reported predictors to capture important dimensions of risk not reflected in health registry data alone. We also developed models based solely on survey data, which yielded C-statistics of 0.73 for single-year high-cost and 0.77 for persistent high-cost users, suggesting that survey-based models may serve as a viable alternative in settings where administrative registry data are not readily available. In line with this, a study predicting high-cost users (top 25%) among privately insured US workers reported C-statistics of 0.78 for a registry-based model and 0.73 for a model based on self-reported health and utilisation data alone [11].

We explored both traditional logistic regression and a machine learning approach, and the discriminatory performance across modelling approaches was largely similar, with XGBoost showing some signs of miscalibration for persistent high-cost prediction. This likely reflects the structured nature of the predictors and the dominant contribution of prior-year healthcare costs and morbidity burden, which are readily captured by conventional regression models. These findings are consistent with previous evidence showing that complex algorithms rarely outperform well-specified statistical models when applied to structured, tabular health data [34]. Although machine learning may offer benefits in more complex data environments, in settings that rely on structured survey and registry data, the clarity, transparency, and usability of traditional statistical models may be preferable.

## Limitations

This study has limitations. Although we used large, population-based samples and performed external validation on individuals from a different geographical region, both cohorts originated from Norway, a setting characterised by universal healthcare coverage and a relatively homogeneous population, which may limit generalisability to more fragmented or privately funded healthcare settings. Within Norway, the two cohorts were generally similar in demographic composition and healthcare utilisation but differed in geography, timing, and baseline health profiles, which may have influenced model performance. The close agreement in discrimination, calibration-in-the-large, and calibration slope between development and external validation cohorts indicates that any overfitting is likely to be limited. However, because both cohorts originate from the same national healthcare system, the validation primarily assesses reproducibility and within-system geographic transportability rather than broader transportability to different healthcare contexts. As such, generalisability to other healthcare systems and international settings remains uncertain [35, 36]. Further, the administrative health registries capture all government-funded healthcare utilisation across primary and secondary care but do not include services from private healthcare providers without a reimbursement contract. In Norway, public healthcare accounts for 99% of inpatient and 86% of outpatient medical care spending [37]. Accordingly, total costs per patient are slightly underestimated. It is also likely that other relevant predictors not available in both surveys could have improved model performance. Finally, we included prior-year healthcare costs as a predictor, as is the case in most existing models [7]. This requires access to administrative registry data and may limit implementation in settings where such data are unavailable. However, the survey-based models demonstrated acceptable performance, with self-reported data on previous healthcare utilisation emerging as significant predictors. This suggests that risk stratification may not only be relevant for healthcare planners but also feasible in clinical contexts lacking access to comprehensive administrative data.

Future research should evaluate the models in non-Norwegian healthcare systems and examine how model-based risk stratification can be implemented in practice. In addition, assessing the cost-effectiveness of interventions guided by these predictions will be an important next step.

## Conclusions

We developed and externally validated multivariable prediction models identifying individuals at risk of single-year and persistent high-cost healthcare use in the general adult population. The models demonstrated good discrimination and calibration across outcomes, with stable performance in external data. While prior-year healthcare costs and morbidity burden were key predictors, the addition of self-reported survey data improved predictive accuracy. Survey-based models alone also yielded acceptable performance, suggesting that risk stratification is feasible even in settings lacking access to administrative health registry data. The findings support the potential utility of prediction models to inform targeted preventive strategies and guide population health management efforts within publicly funded healthcare systems.

## Electronic supplementary material

Below is the link to the electronic supplementary material.

Supplementary material 1

## Data Availability

The datasets generated and/or analysed during the current study are not publicly available because it is governed by Norwegian law, but can be accessed by application to HUNT Research Center and the Tromsø Study Data and Publication Committee.

## Abbreviations

HUNT: Trøndelag Health Study
KUHR: Norwegian Control and Payment of Health Reimbursements Database
NPR: Norwegian Patient Registry
CITL: Calibration-in-the-large
