# Supplementary material for: Predicting high-cost healthcare users: development and external validation of multivariable models using the HUNT and Tromsø studies linked to Norwegian health registries
Source: BMC Health Serv Res. 2026 Mar 6;26:518. doi: 10.1186/s12913-026-14295-7 (PMC13081372; doi:10.1186/s12913-026-14295-7)
Supplement: Supplementary file 1 — Supplementary material 1 [file 12913_2026_14295_MOESM1_ESM.docx]

**Supplementary Table 1.** Description of predictors

| **Variables (functional form)** | **Response categories** | **Cohort differences** |
| --- | --- | --- |
| Sex (binary) | Male or female |  |
| Age at participation (continuous) | In years |  |
| Marital status (categorical) | Unmarried; Married; Widow(er); Divorced |  |
| BMI (continuous) | Kg/m^2^ |  |
| Pulse (continuous) | Beats per minute |  |
| Systolic blood pressure (continuous) | Mean of two measurements, in mm HG |  |
| Diastolic blood pressure (continuous) | Mean of two measurements, in mm HG |  |
| Waist circumference (continuous) | In centimeters |  |
| Hip circumference (continuous) | In centimeters |  |
| Education (categorical) | Primary; Upper secondary (≥3 years); College/University <4 years; College/University ≥4 years |  |
| Current health (categorical) | Poor; Not so good; Good; Very good | Tromsø: Very bad/bad recoded as Poor; Neither good nor bad as Not so good; Excellent as Very good |
| Life satisfaction (categorical) | Very satisfied; Satisfied; Somewhat satisfied; Neither satisfied nor dissatisfied; Somewhat dissatisfied; Dissatisfied or very dissatisfied |  |
| Chronic pain elbows/hands (binary) | No/Yes | HUNT combined question for elbow and hand pain |
| Chronic pain neck (binary) | No/Yes | HUNT combined questions for neck and shoulder pain |
| Chronic pain upper back (binary) | No/Yes |  |
| Chronic pain lumbar back (binary) | No/Yes |  |
| Chronic pain hip/leg (binary) | No/Yes | HUNT combined questions for hip, knee, and ankle pain. |
| Pain intensity | No pain; Mild; Moderate; Severe; Extreme | HUNT: pain in last 4 weeks Tromsø: current pain |
| Spec. consult. last 12 mo. (binary) | No/Yes |  |
| Admitted hospital last 12 mo. (binary) | No/Yes |  |
| Alt. med. consult. last 12 mo. (binary) | No/Yes |  |
| Smoking status (categorical) | Never smoked; Ex occasional smoker; Current occasional smoker; Ex daily smoker; Current daily smoker | Tromsø: Daily and occasional smoking variables combined to match 5-level smoking status |
| Alcohol frequency (categorical) | Never; Monthly or less; 2-4 times a month; 2-3 times a week; 4 or more times a week |  |
| Headache last 12 months (binary) | No/Yes |  |
| HUNT activity index (continuous) | Based on responses about exercise frequency, duration and intensity (range 0-15) |  |
| Type of work (categorical) | Not working; Mostly sedentary work; Requires a lot of walking; Requires you to walk and lift a lot; Heavy manual labour |  |
| Work shifts (binary) | No/Yes |  |
| HADS depression (continuous) | Total score (range 0-21) |  |
| HADS anxiety (continuous) | Total score (range 0-21) |  |
| ICPC morbidity index | Index based on 18 morbidities (range 0-18) |  |
| Healthcare costs preceding 12 mo. | Categorised into quartiles based on the distribution in the full sample (Q1-Q4, lowest to highest costs) |  |

Spec=specialist; Alt. med.=Alternative medicine; HADS=Hospital Anxiety and Depression Scale

**Supplementary Table 2.** Hyperparameters of machine learning models

| **Model and hyperparameter** | **Range explored** | **Selected value** | |
| --- | --- | --- | --- |
|  |  | **Single-year high-cost** | **Persistent high-cost** |
| XGBoost, binary:logistic | | | |
| Learning rate | 0.0001-0.1 | 0.0629 | 0.0038 |
| Max depth | 1, 2, 4, 8 | 2 | 4 |
| N of estimators | 100, 500 | 100 | 500 |
| Gamma | 0, 1, 5, 10 | 5 | 1 |
| Min child weight | 1, 5, 10 | 20 | 10 |
| Colsample bytree | 0.1-0.8 | 0.3871 | 0.6597 |
| Subsample | 0.1, 0.2, 0.3, 0.4, 0.5 | 0.2 | 0.2 |
| Reg alpha | 0, 1, 5, 10, 15, 20 | 5 | 5 |
| Reg lambda | 1, 5, 10, 15, 20 | 15 | 15 |

**Supplementary Table 3.** Number (%) of missing values in the development and external validation cohort

|  | **HUNT4** (development)  (n=42,049) | **Tromsø 7** (validation) (n=20,942) |
| --- | --- | --- |
| Sex | 0 | 0 |
| Age (years) | 0 | 0 |
| Marital status | 81 (0) | 0 |
| Body mass index | 252 (1) | 61 (0) |
| Pulse | 804 (2) | 34 (0) |
| Syst. blood pressure | 105 (0) | 49 (0) |
| Dias. blood pressure | 105 (0) | 73 (0) |
| Waist circumference | 1,286 (3) | 87 (0) |
| Hip circumference | 1,293 (3) | 86 (0) |
| Education | 223 (0.5) | 369 (2) |
| Current health | 581 (1) | 189 (1) |
| Life satisfaction | 450 (1) | 623 (3) |
| Chronic pain elbow/hands | 1,035 (2.5) | 1,432 (7) |
| Chronic pain neck | 1,035 (2.5) | 945 (5) |
| Chronic pain upper back | 1,035 (2.5) | 1,898 (9) |
| Chronic pain lumbar back | 1,035 (2.5) | 1,345 (6) |
| Chronic pain hip/leg | 1,035 (2.5) | 1,075 (5) |
| Pain intensity | 665 (2) | 436 (2) |
| Spec. consultation last 12 mo. | 3,362 (8) | 942 (5) |
| Admitted hospital last 12 mo. | 515 (1) | 206 (1) |
| Alt. med. consultation last 12 mo. | 2,758 (7) | 682 (3) |
| Smoking status | 192 (1) | 152 (1) |
| Alcohol frequency | 556 (1) | 128 (1) |
| Headache last 12 mo. | 1,214 (3) | 367 (2) |
| HUNT act. index (range 0-15) | 1,143 (3) | 661 (3) |
| Type of work | 634 (2) | 507 (2) |
| Works shifts | 503 (1) | 973 (5) |
| HADS depression (range 0-21) | 1,193 (3) | 1,220 (6) |
| HADS anxiety (range 0-21) | 1,491 (4) | 1,566 (8) |
| ICPC morbidity index | 0 | 0 |
| Costs preceding 12 mo. | 0 | 0 |

Syst=systolic; Dias=diastolic; Spec=specialist; Alt. med.=Alternative medicine; HADS=Hospital Anxiety and Depression Scale

**Supplementary Table 4.** Coefficients and odds ratios from logistic regression models using survey and registry data

| **Parameter** | **Single-year high-cost** | | | **Persistent high-cost** | | |
| --- | --- | --- | --- | --- | --- | --- |
|  | Coef. | OR (95% CI) | Shrunken coef. | Coef. | OR (95% CI) | Shrunken coef. |
| Sex (ref: male) | 0 | 1.00 | 0 | 0 | 1.00 | 0 |
| Female | -0.111 | 0.90 (0.84, 0.96) | -0.0039 | -0.064 | 0.94 (0.86, 1.02) | -0.0028 |
| Age (continuous) | - | - | - | - | - | - |
| 1^st^ FP term | 0.001 | 1.00 (1.00, 1.00) | 0.0001 | 0.013 | 1.01 (1.01, 1.02) | 0.0001 |
| Marital status (ref: Unmarried) | 0 | 1.00 | 0 | 0 | 1.00 | 0 |
| Married | -0.097 | 0.91 (0.85, 0.97) | -0.0035 | -0.117 | 0.89 (0.81, 0.98) | -0.0057 |
| Widow(er) | -0.117 | 0.89 (0.79, 1.01) | -0.0073 | -0.136 | 0.87 (0.75, 1.01) | -0.0104 |
| Divorced | -0.051 | 0.95 (0.86, 1.04) | -0.0025 | -0.041 | 0.96 (0.85, 1.08) | -0.0026 |
| BMI (continuous) | - | - | - | 0.001 | 1.00 (0.98, 1.03) | 0.0001 |
| 1^st^ FP term | 0.036 | 1.04 (1.00, 1.07) | 0.0006 | - | - | - |
| 2^st^ FP term | -0.020 | 0.98 (0.96, 1.00) | -0.0002 | - | - | - |
| Pulse (continuous) | 0.004 | 1.00 (1.00, 1.01) | 0.0001 | 0.003 | 1.00 (1.00, 1.01) | 0.0001 |
| Syst. blood pressure (continuous) | - | - | - | - | - | - |
| 1^st^ FP term | 0.522 | 1.68 (1.21, 2.34) | 0.0871 | 0.649 | 1.91 (1.28, 2.87) | 0.1340 |
| 2 ^st^ FP term | 0.148 | 1.16 (1.03, 1.31) | 0.0091 | 0.203 | 1.22 (1.06, 1.42) | 0.0152 |
| Dias. blood pressure (continuous) | -0.003 | 1.00 (0.99, 1.00) | 0.0000 | -0.003 | 1.00 (0.99, 1.00) | 0.0001 |
| Waist circumference (continuous) | -0.003 | 1.00 (0.99, 1.00) | 0.0000 | -0.002 | 1.00 (0.99, 1.00) | 0.0001 |
| Hip circumference (continuous) | 0.000 | 1.00 (0.99, 1.01) | 0.0000 | 0.009 | 1.01 (0.99, 1.02) | 0.0001 |
| Education (ref: Primary School) | 0 | 1.00 | 0 | 0 | 1.00 | 0 |
| Upper secondary school | 0.032 | 1.03 (0.97, 1.10) | 0.0011 | 0.063 | 1.06 (0.98, 1.16) | 0.0027 |
| University <4 years | 0.095 | 1.10 (1.02, 1.19) | 0.0038 | 0.120 | 1.13 (1.02, 1.24) | 0.0060 |
| University 4 years or more | 0.108 | 1.11 (1.02, 1.21) | 0.0046 | 0.124 | 1.13 (1.02, 1.26) | 0.0068 |
| Current health (ref: Poor) | 0 | 1.00 | 0 | 0 | 1.00 | 0 |
| Not so good | -0.174 | 0.84 (0.69, 1.02) | -0.0176 | 0.042 | 1.04 (0.85, 1.27) | 0.0043 |
| Good | -0.537 | 0.58 (0.48, 0.72) | -0.0559 | -0.348 | 0.71 (0.57, 0.87) | -0.0377 |
| Very good | -0.799 | 0.45 (0.36, 0.56) | -0.0916 | -0.739 | 0.48 (0.37, 0.62) | -0.0976 |
| Life satisfaction (ref: Very satisfied) | 0 | 1.00 | 0 | 0 | 1.00 | 0 |
| Satisfied | -0.045 | 0.96 (0.88, 1.04) | -0.0019 | 0.045 | 1.05 (0.94, 1.17) | 0.0026 |
| Somewhat satisfied | -0.058 | 0.94 (0.87, 1.03) | -0.0025 | 0.043 | 1.04 (0.93, 1.17) | 0.0025 |
| Neither satisfied nor dissatisfied | -0.001 | 1.00 (0.90, 1.11) | -0.0001 | 0.158 | 1.17 (1.02, 1.34) | 0.0110 |
| Somewhat dissatisfied | 0.204 | 1.23 (1.00, 1.50) | 0.0212 | 0.310 | 1.36 (1.07, 1.73) | 0.0379 |
| Dissatisfied or very dissatisfied | 0.156 | 1.17 (0.90, 1.52) | 0.0207 | 0.475 | 1.61 (1.20, 2.16) | 0.0710 |
| Chronic pain elbows/hands (ref: No) | 0 | 1.00 | 0 | 0 | 1.00 | 0 |
| Yes | -0.008 | 0.99 (0.93, 1.06) | -0.0003 | 0.028 | 1.03 (0.95, 1.12) | 0.0012 |
| Chronic pain neck (ref: No) | 0 | 1.00 | 0 | 0 | 1.00 | 0 |
| Yes | 0.063 | 1.06 (1.00, 1.14) | 0.0021 | 0.062 | 1.06 (0.98, 1.15) | 0.0026 |
| Chronic pain upper back (ref: No) | 0 | 1.00 | 0 | 0 | 1.00 | 0 |
| Yes | 0.036 | 1.04 (0.96, 1.12) | 0.0015 | 0.118 | 1.13 (1.02, 1.24) | 0.0058 |
| Chronic pain lumbar back (ref: No) | 0 | 1.00 | 0 | 0 | 1.00 | 0 |
| Yes | 0.030 | 1.03 (0.96, 1.10) | 0.0010 | 0.093 | 1.10 (1.01, 1.19) | 0.0038 |
| Chronic pain hip/leg (ref: No) | 0 | 1.00 | 0 | 0 | 1.00 | 0 |
| Yes | 0.059 | 1.06 (1.00, 1.13) | 0.0019 | 0.078 | 1.08 (1.00, 1.17) | 0.0031 |
| Pain intensity (ref: No pain) | 0 | 1.00 | 0 | 0 | 1.00 | 0 |
| Mild | 0.042 | 1.04 (0.97, 1.12) | 0.0016 | 0.099 | 1.10 (0.99, 1.23) | 0.0054 |
| Moderate | 0.280 | 1.32 (1.22, 1.44) | 0.0119 | 0.293 | 1.34 (1.20, 1.50) | 0.0171 |
| Severe | 0.602 | 1.83 (1.64, 2.03) | 0.0330 | 0.583 | 1.79 (1.56, 2.05) | 0.0405 |
| Extreme | 0.760 | 2.14 (1.72, 2.67) | 0.0856 | 0.790 | 2.20 (1.73, 2.80) | 0.0968 |
| Spec. consult. last 12 mo (ref: No) | 0 | 1.00 | 0 | 0 | 1.00 | 0 |
| Yes | 0.174 | 1.19 (1.10, 1.28) | 0.0066 | 0.090 | 1.09 (1.00, 1.20) | 0.0041 |
| Admitted hospital last 12 mo (ref: No) | 0 | 1.00 | 0 | 0 | 1.00 | 0 |
| Yes | -0.218 | 0.80 (0.75, 0.87) | -0.0085 | -0.195 | 0.82 (0.76, 0.89) | -0.0083 |
| Alt. med. Consult. last 12 mo (ref: No) | 0 | 1.00 | 0 | 0 | 1.00 | 0 |
| Yes | 0.021 | 1.02 (0.91, 1.14) | 0.0012 | 0.043 | 1.04 (0.91, 1.20) | 0.0030 |
| Smoking status (ref: Never smoked) | 0 | 1.00 | 0 | 0 | 1.00 | 0 |
| Ex-occasional smoker | 0.075 | 1.08 (0.98, 1.19) | 0.0037 | -0.037 | 0.96 (0.85, 1.10) | -0.0025 |
| Current occasional smoker | 0.057 | 1.06 (0.82, 1.36) | 0.0073 | 0.236 | 1.27 (0.91, 1.76) | 0.0398 |
| Ex-daily smoker | 0.123 | 1.13 (1.07, 1.20) | 0.0037 | 0.073 | 1.08 (1.00, 1.16) | 0.0027 |
| Current daily smoker | -0.034 | 0.97 (0.88, 1.07) | -0.0017 | -0.140 | 0.87 (0.77, 0.98) | -0.0089 |
| Alcohol frequency (ref: Never) | 0 | 1.00 | 0 | 0 | 1.00 | 0 |
| Monthly or less | 0.049 | 1.05 (0.96, 1.14) | 0.0022 | 0.101 | 1.11 (1.00, 1.23) | 0.0053 |
| 2-4 times a month | -0.080 | 0.92 (0.85, 1.00) | -0.0034 | -0.013 | 0.99 (0.89, 1.10) | -0.0007 |
| 2-3 times a week | -0.039 | 0.96 (0.87, 1.06) | -0.0019 | -0.008 | 0.99 (0.88, 1.12) | -0.0005 |
| 4 times or more a week | -0.082 | 0.92 (0.78, 1.08) | -0.0067 | 0.025 | 1.03 (0.84, 1.25) | 0.0026 |
| Headache last month (ref: No) | 0 | 1.00 | 0 | 0 | 1.00 | 0 |
| Yes | -0.008 | 0.99 (0.93, 1.05) | -0.0002 | 0.067 | 1.07 (0.99, 1.15) | 0.0026 |
| HUNT activity index (continuous) | - | - | - | 0.002 | 1.00 (0.99, 1.02) | 0.0001 |
| 1^st^ FP term | 0.000 | 1.00 (1.00, 1.00) | 0.0000 | - | - | - |
| Type of work (ref: Not working) | 0 | 1.00 | 0 | 0 | 1.00 | 0 |
| Mostly sedentary | -0.180 | 0.84 (0.77, 0.91) | -0.0080 | -0.345 | 0.71 (0.63, 0.79) | -0.0204 |
| Requires a lot of walking | -0.048 | 0.95 (0.87, 1.04) | -0.0022 | -0.174 | 0.84 (0.75, 0.94) | -0.0102 |
| Require you to walk and lift | -0.090 | 0.91 (0.83, 1.00) | -0.0043 | -0.165 | 0.85 (0.75, 0.96) | -0.0103 |
| Heavy manual labour | -0.133 | 0.88 (0.75, 1.03) | -0.0108 | -0.467 | 0.63 (0.50, 0.79) | -0.0539 |
| Work shifts (ref: No) | 0 | 1.00 | 0 | 0 | 1.00 | 0 |
| Yes | 0.016 | 1.02 (0.93, 1.11) | 0.0007 | -0.035 | 0.97 (0.86, 1.09) | -0.0021 |
| HADS depression (continuous) | - | - | - | - | - | - |
| 1^st^ FP term | -0.021 | 0.98 (0.92, 1.04) | -0.0006 | -0.141 | 0.87 (0.79, 0.96) | -0.0072 |
| HADS anxiety (continuous) | - | - | - | - | - | - |
| 1^st^ FP term | -0.003 | 1.00 (0.94, 1.06) | 0.0001 | 0.061 | 1.06 (0.99, 1.14) | 0.0023 |
| ICPC morbidity index (continuous) | 0.347 | 1.42 (1.33, 1.50) | 0.0107 | 0.446 | 1.56 (1.46, 1.67) | 0.0151 |
| Cost category (ref: Quartile 1) | 0 | 1.00 | 0 | 0 | 1.00 | 0 |
| Quartile 2 | 0.536 | 1.71 (1.55, 1.88) | 0.0267 | 0.632 | 1.88 (1.58, 2.24) | 0.0561 |
| Quartile 3 | 1.199 | 3.32 (3.02, 3.64) | 0.0570 | 1.390 | 4.01 (3.41, 4.72) | 0.1147 |
| Quartile 4 | 2.214 | 9.16 (8.29, 10.11) | 0.1119 | 2.686 | 14.68 (12.49, 17.25) | 0.2211 |
| Constant | -1.861 |  | -1.1656 | -4.561 |  | -2.2829 |

Syst=systolic; Dias=diastolic; Spec=specialist; Alt. med.=Alternative medicine; HADS=Hospital Anxiety and Depression Scale
Fractional polynomial terms:
High-cost model: age_fp1 = (x^3)-165.387, x = age/10
 bmi_fp1 = (x^3)-20.251, fp2 = ((x^3)*ln(x))-20.307, x = bmi/10
 bpsyst_fp1 = (x^-2)-0.603, fp2 = ((x^2)*ln(x))-.153, x = bpsyst/100
 actindex_fp1 = (x^-2)-9.7570, x = (actindex+.005)/100
 hadsdep_fp1 = (x^3)-0.076, x = (hadsdep+1)/10
 hadsanx_fp1 = (x^2)-0.291, x = (hadsanx+1)/10
Persistent model: age_fp1 = (x^2)-30.130, age_x = age/10
 bpsyst_fp1 = (x^-2)-0.603, fp2 = ((x^2)*ln(x))-.153, x = bpsyst/100
 hadsdep_fp1 = (x^2)-0.179, x = (hadsdep+1)/10
 hadsanx_fp1 = (x^2)-0.291, x = (hadsanx+1)/10

**Supplementary Table 5.** Coefficients and odds ratios from logistic regression models using only survey data

| **Parameter** | **Single-year high-cost** | | | **Persistent high-cost** | | |
| --- | --- | --- | --- | --- | --- | --- |
|  | Coef. | OR (95% CI) | Shrunken coef. | Coef. | OR (95% CI) | Shrunken coef. |
| Sex (ref: male) | 0 | 1.00 | 0 | 0 | 1.00 | 0 |
| Female | -0.165 | 0.85 (0.79, 0.91) | -0.0055 | -0.092 | 0.91 (0.84, 0.99) | -0.0039 |
| Age (continuous) | - | - | - | - | - | - |
| 1^st^ FP term | 0.002 | 1.00 (1.00, 1.00) | 0.0001 | 0.019 | 1.02 (1.02, 1.02) | 0.0000 |
| Marital status (ref: Unmarried) | 0 | 1.00 | 0 | 0 | 1.00 | 0 |
| Married | -0.052 | 0.95 (0.89, 1.02) | -0.0018 | -0.078 | 0.92 (0.84, 1.01) | -0.0036 |
| Widow(er) | -0.108 | 0.90 (0.80, 1.01) | -0.0065 | -0.119 | 0.89 (0.77, 1.03) | -0.0087 |
| Divorced | 0.005 | 1.01 (0.92, 1.10) | 0.0002 | 0.002 | 1.00 (0.89, 1.13) | 0.0001 |
| BMI (continuous) | 0.016 | 1.02 (1.00, 1.04) | 0.0002 | 0.014 | 1.01 (0.99, 1.04) | 0.0002 |
| Pulse (continuous) | 0.005 | 1.01 (1.00, 1.01) | 0.0001 | 0.005 | 1.01 (1.00, 1.01) | 0.0001 |
| Syst. blood pressure (continuous) | - | - | - | - | - | - |
| 1^st^ FP term | 0.643 | 1.90 (1.39, 2.60) | 0.1029 | 0.784 | 2.19 (1.49, 3.22) | 0.1545 |
| 2^st^ FP term | 0.125 | 1.13 (1.01, 1.27) | 0.0073 | 0.188 | 1.21 (1.05, 1.39) | 0.0133 |
| Dias. blood pressure (continuous) | -0.004 | 1.00 (0.99, 1.00) | 0.0001 | -0.005 | 1.00 (0.99, 1.00) | 0.0000 |
| Waist circumference (continuous) | -0.002 | 1.00 (0.99, 1.00) | 0.0001 | -0.001 | 1.00 (0.99, 1.00) | 0.0000 |
| Hip circumference (continuous) | 0.000 | 1.00 (0.99, 1.01) | 0.0001 | -0.299 | 0.74 (0.20, 2.75) | -0.1996 |
| Education (ref: Primary School) | 0 | 1.00 | 0 | 0 | 1.00 | 0 |
| Upper secondary school | 0.030 | 1.03 (0.97, 1.10) | 0.0010 | 0.059 | 1.06 (0.98, 1.15) | 0.0024 |
| University <4 years | 0.115 | 1.12 (1.04, 1.21) | 0.0044 | 0.137 | 1.15 (1.04, 1.26) | 0.0066 |
| University 4 years or more | 0.124 | 1.13 (1.04, 1.23) | 0.0051 | 0.151 | 1.16 (1.05, 1.29) | 0.0079 |
| Current health (ref: Poor) | 0 | 1.00 | 0 | 0 | 1.00 | 0 |
| Not so good | -0.253 | 0.78 (0.64, 0.94) | -0.0248 | -0.079 | 0.92 (0.76, 1.12) | -0.0078 |
| Good | -0.822 | 0.44 (0.36, 0.54) | -0.0829 | -0.723 | 0.49 (0.40, 0.60) | -0.0758 |
| Very good | -1.257 | 0.28 (0.23, 0.35) | -0.1394 | -1.352 | 0.26 (0.20, 0.33) | -0.1723 |
| Life satisfaction (ref: Very satisfied) | 0 | 1.00 | 0 | 0 | 1.00 | 0 |
| Satisfied | -0.055 | 0.95 (0.88, 1.02) | -0.0022 | 0.025 | 1.03 (0.92, 1.14) | 0.0014 |
| Somewhat satisfied | -0.073 | 0.93 (0.86, 1.01) | -0.0031 | 0.023 | 1.02 (0.92, 1.14) | 0.0013 |
| Neither satisfied nor dissatisfied | 0.026 | 1.03 (0.93, 1.14) | 0.0013 | 0.182 | 1.20 (1.05, 1.37) | 0.0122 |
| Somewhat dissatisfied | 0.257 | 1.29 (1.06, 1.57) | 0.0256 | 0.374 | 1.45 (1.16, 1.83) | 0.0437 |
| Dissatisfied or very dissatisfied | 0.218 | 1.24 (0.97, 1.60) | 0.0278 | 0.537 | 1.71 (1.29, 2.27) | 0.0770 |
| Chronic pain elbows/hands (ref: No) | 0 | 1.00 | 0 | 0 | 1.00 | 0 |
| Yes | -0.001 | 1.00 (0.94, 1.07) | 0.0001 | 0.022 | 1.02 (0.95, 1.10) | 0.0009 |
| Chronic pain neck (ref: No) | 0 | 1.00 | 0 | 0 | 1.00 | 0 |
| Yes | 0.121 | 1.13 (1.06, 1.20) | 0.0039 | 0.122 | 1.13 (1.04, 1.22) | 0.0049 |
| Chronic pain upper back (ref: No) | 0 | 1.00 | 0 | 0 | 1.00 | 0 |
| Yes | 0.109 | 1.12 (1.03, 1.21) | 0.0044 | 0.188 | 1.21 (1.10, 1.32) | 0.0088 |
| Chronic pain lumbar back (ref: No) | 0 | 1.00 | 0 | 0 | 1.00 | 0 |
| Yes | 0.044 | 1.04 (0.98, 1.11) | 0.0014 | 0.102 | 1.11 (1.03, 1.20) | 0.0040 |
| Chronic pain hip/leg (ref: No) | 0 | 1.00 | 0 | 0 | 1.00 | 0 |
| Yes | 0.098 | 1.10 (1.04, 1.17) | 0.0030 | 0.112 | 1.12 (1.04, 1.21) | 0.0044 |
| Pain intensity (ref: No pain) | 0 | 1.00 | 0 | 0 | 1.00 | 0 |
| Mild | 0.064 | 1.07 (0.99, 1.15) | 0.0024 | 0.118 | 1.13 (1.02, 1.25) | 0.0061 |
| Moderate | 0.348 | 1.42 (1.31, 1.53) | 0.0142 | 0.365 | 1.44 (1.29, 1.61) | 0.0203 |
| Severe | 0.707 | 2.03 (1.83, 2.25) | 0.0370 | 0.693 | 2.00 (1.76, 2.28) | 0.0461 |
| Extreme | 0.888 | 2.43 (1.96, 3.01) | 0.0974 | 0.913 | 2.49 (1.97, 3.15) | 0.1087 |
| Spec. consult. last 12 mo (ref: No) | 0 | 1.00 | 0 | 0 | 1.00 | 0 |
| Yes | 0.443 | 1.56 (1.45, 1.67) | 0.0160 | 0.370 | 1.45 (1.33, 1.58) | 0.0162 |
| Admitted hospital last 12 mo (ref: No) | 0 | 1.00 | 0 | 0 | 1.00 | 0 |
| Yes | 0.787 | 2.20 (2.06, 2.34) | 0.0257 | 0.875 | 2.40 (2.23, 2.58) | 0.0326 |
| Alt. med. Consult. last 12 mo (ref: No) | 0 | 1.00 | 0 | 0 | 1.00 | 0 |
| Yes | 0.043 | 1.04 (0.94, 1.16) | 0.0024 | 0.051 | 1.05 (0.92, 1.20) | 0.0034 |
| Smoking status (ref: Never smoked) | 0 | 1.00 | 0 | 0 | 1.00 | 0 |
| Ex-occasional smoker | 0.083 | 1.09 (0.99, 1.19) | 0.0039 | -0.013 | 0.99 (0.87, 1.12) | -0.0008 |
| Current occasional smoker | 0.012 | 1.01 (0.79, 1.29) | 0.0015 | 0.192 | 1.21 (0.89, 1.65) | 0.0305 |
| Ex-daily smoker | 0.175 | 1.19 (1.13, 1.26) | 0.0050 | 0.136 | 1.15 (1.07, 1.23) | 0.0049 |
| Current daily smoker | -0.055 | 0.95 (0.86, 1.04) | -0.0026 | -0.152 | 0.86 (0.76, 0.97) | -0.0092 |
| Alcohol frequency (ref: Never) | 0 | 1.00 | 0 | 0 | 1.00 | 0 |
| Monthly or less | 0.006 | 1.01 (0.93, 1.09) | 0.0002 | 0.045 | 1.05 (0.95, 1.16) | 0.0023 |
| 2-4 times a month | -0.145 | 0.86 (0.80, 0.94) | -0.0060 | -0.107 | 0.90 (0.81, 0.99) | -0.0055 |
| 2-3 times a week | -0.121 | 0.89 (0.81, 0.97) | -0.0059 | -0.114 | 0.89 (0.79, 1.01) | -0.0069 |
| 4 times or more a week | -0.128 | 0.88 (0.75, 1.03) | -0.0100 | -0.048 | 0.95 (0.79, 1.15) | -0.0047 |
| Headache last month (ref: No) | 0 | 1.00 | 0 | 0 | 1.00 | 0 |
| Yes | -0.001 | 1.00 (0.94, 1.06) | 0.0001 | 0.067 | 1.07 (0.99, 1.15) | 0.0025 |
| HUNT activity index (continuous) | - | - | - | - | - | - |
| 1^st^ FP term | 0.000 | 1.00 (1.00, 1.00) | 0.0000 | -0.002 | 1.00 (1.00, 1.00) | 0.0000 |
| 2^st^ FP term | 0.000 | 1.00 (1.00, 1.00) | 0.0000 | - | - |  |
| Type of work (ref: Not working) | 0 | 1.00 | 0 | 0 | 1.00 | 0 |
| Mostly sedentary | -0.272 | 0.76 (0.70, 0.83) | -0.0116 | -0.449 | 0.64 (0.57, 0.71) | -0.0254 |
| Requires a lot of walking | -0.121 | 0.89 (0.81, 0.96) | -0.0052 | -0.254 | 0.78 (0.69, 0.87) | -0.0143 |
| Require you to walk and lift | -0.164 | 0.85 (0.78, 0.93) | -0.0075 | -0.258 | 0.77 (0.69, 0.87) | -0.0154 |
| Heavy manual labour | -0.179 | 0.84 (0.72, 0.97) | -0.0140 | -0.508 | 0.60 (0.48, 0.75) | -0.0566 |
| Work shifts (ref: No) | 0 | 1.00 | 0 | 0 | 1.00 | 0 |
| Yes | -0.001 | 1.00 (0.92, 1.09) | 0.0001 | -0.053 | 0.95 (0.84, 1.07) | -0.0031 |
| HADS depression (continuous) | - | - | - | - | - | - |
| 1^st^ FP term | -0.023 | 0.98 (0.92, 1.03) | -0.0007 | -0.126 | 0.88 (0.80, 0.97) | -0.0061 |
| HADS anxiety (continuous) | - | - | - | - | - | - |
| 1^st^ FP term | -0.011 | 0.99 (0.93, 1.05) | -0.0003 | 0.034 | 1.03 (0.97, 1.11) | 0.0012 |
| Constant | -1.275 |  | -0.5088 | -2.348 |  | -0.8777 |

Syst=systolic; Dias=diastolic; Spec=specialist; Alt. med.=Alternative medicine; HADS=Hospital Anxiety and Depression Scale
Fractional polynomial terms:
High-cost model: age_fp1 = (x^3)-165.387, x = age/10
 bpsyst_fp1 = (x^-2)-0.603, bpsyst_fp2 = ((x^2)*ln(x))-.153, x = bpsyst/100
 actindex_fp1 = (x^-2)-9.757, actindex_fp2 = ((x^-2)*ln(x))+11.113, x = (actindex+.005)/100
 hadsdep_fp1 = (hadsdep_x^3)-0.076, hadsdep_x = (hadsdep+1)/10
 hadsanx_fp1 = (hadsanx_x^2)-0.291, hadsanx_x = (hadsanx+1)/10
Persistent model: age_fp1 = (x^2)-30.130, age_x = age/10
 bpsyst_fp1 = (x^-2)-0.603, fp2 = ((x^2)*ln(x))-.153, x = bpsyst/100
 hipcirc_fp1 = (x^-1)-0.989, x = hipcirc/100
 actindex_fp1 = (x^-2)-9.757, x= (actindex+1)/10
 hadsdep_fp1 = (x^2)-0.179, x = (hadsdep+1)/10
 hadsanx_fp1 = (x^2)-0.291, x = (hadsanx+1)/10

**Supplementary Table 6.** Coefficients and odds ratios from logistic regression models using age, gender and registry data

| **Parameter** | **Single-year high-cost** | | | **Persistent high-cost** | | |
| --- | --- | --- | --- | --- | --- | --- |
|  | Coef. | OR (95% CI) | Shrunken coef. | Coef. | OR (95% CI) | Shrunken coef. |
| Sex (ref: male) | 0 | 1.00 | 0 | 0 | 1.00 | 0 |
| Female | -0.245 | 0.78 (0.74, 0.82) | -0.0063 | -0.229 | 0.80 (0.75, 0.85) | -0.0076 |
| Age | 0.011 | 1.01 (1.01, 1.01) | 0 | 0.015 | 1.02 (1.01, 1.02) | 0.0000 |
| ICPC morbidity index (continuous) | 0.435 | 1.55 (1.46, 1.64) | 0.0128 | 0.523 | 1.69 (1.58, 1.80) | 0.0168 |
| Cost category (ref: Quartile 1) | 0 | 1.00 | 0 | 0 | 1.00 | 0 |
| Quartile 2 | 0.662 | 1.94 (1.76, 2.13) | 0.0325 | 0.798 | 2.22 (1.87, 2.64) | 0.0703 |
| Quartile 3 | 1.474 | 4.37 (3.99, 4.78) | 0.0677 | 1.747 | 5.74 (4.90, 6.72) | 0.1413 |
| Quartile 4 | 2.508 | 12.28 (11.25, 13.42) | 0.1131 | 3.079 | 21.73 (18.66, 25.32) | 0.2399 |
| Constant | -3.106 |  | -0.1771 | -4.693 |  | -0.4314 |

**Supplementary Table 6.** Model performance for predicting single-year high-cost and persistent high-cost patients in subgroups

|  | **Development cohort (n=42,049)** | | |  | **External validation cohort (n=20,942)** | | |
| --- | --- | --- | --- | --- | --- | --- | --- |
|  | C-statistic | CITL | C-slope |  | C-statistic | CITL | C-slope |
| **Single-year high-cost** |  |  |  |  |  |  |  |
| **Female** |  |  |  |  |  |  |  |
| Logistic regression | 0.78 (0.77, 0.79) | 0.00 (-0.03, 0.03) | 1.00 (0.97, 1.03) |  | 0.78 (0.77, 0.79) | 0.01 (-0.04, 0.06) | 0.99 (0.95, 1.04) |
| XGBoost | 0.77 (0.76, 0.78 | 0.05 (0.02, 0.08) | 1.12 (1.08, 1.16) |  | 0.77 (0.76, 0.78) | 0.05 (0.00, 0.10) | 1.17 (1.11, 1.33) |
| **Male** |  |  |  |  |  |  |  |
| Logistic regression | 0.80 (0.79, 0.80) | 0.00 (-0.04, 0.03) | 1.02 (0.99, 1.06) |  | 0.77 (0.76, 0.78) | 0.08 (0.03, 0.13) | 0.96 (0.91, 1.01) |
| XGBoost | 0.79 (0.78, 0.80) | -0.06 (-0.10, -0.02) | 1.19 (1.15, 1.24) |  | 0.76 (0.75, 0.78) | -0.01  (-0.06, 0.04) | 1.14 (1.08, 1.20) |
| **Persistent high-cost** |  |  |  |  |  |  |  |
| **Female** |  |  |  |  |  |  |  |
| Logistic regression | 0.83 (0.82, 0.83) | 0.00 (-0.04, 0.04) | 1.00 (0.96, 1.03) |  | 0.82 (0.81, 0.83) | 0.05 (-0.01, 0.11) | 0.98 (0.93, 1.03) |
| XGBoost | 0.82 (0.81, 0.83) | -0.01 (-0.05, 0.03) | 1.52 (1.47, 1.58) |  | 0.81 (0.80, 0.82) | 0.07 (0.02, 0.12) | 1.52 (1.45, 1.60) |
| **Male** |  |  |  |  |  |  |  |
| Logistic regression | 0.85 (0.84, 0.85) | 0.00 (-0.05, 0.05) | 1.02 (0.98, 1.06) |  | 0.82 (0.81, 0.84) | 0.00 (-0.07, 0.07) | 0.97 (0.91, 1.02) |
| XGBoost | 0.84 (0.83, 0.85) | -0.17 (-0.22, -0.12) | 1.61 (1.55, 1.68) |  | 0.82 (0.80, 0.83) | -0.21 (-0.28, -0.15) | 1.55 (1.46, 1.64) |

CITL=Calibration-in-the-large; C-slope=Calibration slope

**Supplementary Table 7.** Model performance in sensitivity analysis for predicting single-year high-cost and persistent high-cost patients defined based on the top 10th percentile

|  | **Development cohort (n=42,049)** | | |  | **External validation cohort (n=20,942)** | | |
| --- | --- | --- | --- | --- | --- | --- | --- |
|  | C-statistic | CITL | C-slope |  | C-statistic | CITL | C-slope |
| **Single-year high-cost** |  |  |  |  |  |  |  |
| Logistic regression | 0.77 (0.76, 0.77) | 0.00 (-0.03, 0.03) | 1.01 (0.97, 1.04) |  | 0.74 (0.73, 0.75) | 0.08 (0.03, 0.13) | 0.95 (0.90, 1.00) |
| XGBoost | 0.74 (0.73, 0.75) | -0.10 (-0.13, -0.07) | 1.84 (1.77, 1.90) |  | 0.72 (0.71, 0.74) | -0.07 (-0.12, -0.03) | 1.70 (1.61, 1.80) |
| **Persistent high-cost** |  |  |  |  |  |  |  |
| Logistic regression | 0.83 (0.82, 0.84) | 0.00 (-0.06, 0.06) | 1.01 (0.96, 1.06) |  | 0.81 (0.80, 0.83) | 0.16 (0.08, 0.23) | 1.01 (0.94, 1.08) |
| XGBoost | 0.80 (0.79, 0.81) | -0.38 (-0.43, -0.32) | 2.43 (2.31, 2.56) |  | 0.81 (0.79, 0.82) | -0.27 (-0.35, -0.20) | 2.47 (2.29, 2.64) |

CITL=Calibration-in-the-large; C-slope=Calibration slope
